# Hollow Glass Microspheres (HGMs): Synthesis, Characterization, and Processes in Biomedical Applications—A Review

**DOI:** 10.3390/ph19081183

**Published:** 2026-07-28

**Authors:** Olusegun Adigun Afolabi, Ndivhuwo Ndou

**Affiliations:** Department of Industrial Engineering and Engineering Management, University of South Africa, Florida, Johannesburg 1710, South Africa

**Keywords:** hollow glass microspheres, tissue engineering, drug delivery, composite materials, characterization, fabrication

## Abstract

Hollow glass microspheres, as demonstrated in recent studies, have shown significant importance in the field of composite materials and have emerged as transformative materials in biomedical applications. This is necessitated by their ability to provide a physicochemical gradient, a desirable tool for complex tissues and biological interfaces, through the spatiotemporal release of bioactive factors and nanophase ceramics. HGMs are structures with diameters ranging from 1 to 1000 µm that can be used as support for cell growth, either in the form of a scaffold or a drug delivery system. In this review, we describe the various methods for HGM fabrications, synthesis (e.g., flame spraying, sol-gel processes, spray drying, etc.), structural characterizations, and chemical and physical properties (e.g., densities ranging from 0.1 to 0.6 g/cm^3^ and compressive strength ranging from 10 MPa to 30 MPa for low and high densities, respectively), highlighting how these methods influence their drug delivery, tissue engineering, bone implants, and nanocarrier abilities. Furthermore, a comprehensive list of other materials and their various biomedical uses is reported. Some of the limitations of existing techniques and future investigations into how HGM can perform as a biomedical material are discussed.

## 1. Introduction

Hollow glass microspheres (HGMs) are spherical particles typically ranging in size from tens to hundreds of micrometers. They are characterized by a hollow interior surrounded by a thin, glassy shell. HGMs are often fabricated from silica-based materials; however, other compositions, such as borosilicate glass, may also be used [1]. Microspheres are spherical particles that can be classified into two categories: solid or hollow. They typically range in diameter from 1 to 1000 µm and are made from materials such as glass, ceramic, carbon, or plastic, depending on the specific application. Solid glass microspheres (SGMs) are manufactured by direct burning of glass powders, while hollow ones (HGMs) are produced by adding a blowing (bubbling) agent to glass powder [2,3,4].

Hollow glass microspheres (HGMs) are tiny spheres with a high ball-type rate (or their glass bead nature and ball-bearing effect) that can improve mobility and reduce the viscosity and internal stress of the resin mixture. They can be used to give reflectivity and a smooth surface. They are widely used in consumer goods, medicine, research, aerospace, automobiles, and appliances. The incorporation of HGMs into the matrix has a considerable impact on the weight of polymer materials, making them appropriate for a variety of industrial and structural applications in aircraft, maritime, and building partitioning [5]. Glass microspheres have been employed for at least 100 years, with solid glass beads produced in New York as far back as 1914 [4].

In 1922, a considerable amount of high-refractive-index glass beads was fabricated for coating movie screens. In the 1950s, hollow glass microsphere technology was developed [3]. Since then, many sectors have come to depend on SGMs and HGMs as a main constituent in their products and production processes. In the 1960s, they were at first employed as fillers for plastics and found opportunities in a countless number of applications, such as aerospace and military materials, molded plastic components, retro-reflective main road marks, oil and gas, recreation, paints and coatings, transportation, building, mining blasting materials, individual care, cosmetics, etc. [2,6,7,8].

In 1963, Beck created a material called hollow beads, which 3 M later referred to as hollow glass microspheres after reheating the particles to form a single wall. The HGMs were patented in collaboration with Standard Oil of Ohio (Sohio), which was later acquired by Philadelphia Quartz (PQ), now known as PQ Corporation. Hollow glass microspheres (HGMs) are microscopic glass spheres manufactured for a wide range of uses in research, medicine, consumer goods, and various industries. The use of HGMs has extensively revolutionized the applications of polymer matrices with glass microspheres, which are typically between 1 and 1000 micrometers in diameter. The densities of HGMs range from 0.1 to 0.6 g/cm^3^, depending on the wall thickness and size, while solid glass microspheres range from 2.4 to 2.6 g/cm^3^ in density because of their bulk density. The lower density of HGMs makes them lighter by about 4–20 times than solid glass microspheres. This enhances their application for lower or scaffold weight in biomedical devices [2,6,8,9].

Hollow glass microspheres, sometimes referred to as microballoons, have diameters ranging from 1 to 1000 µm. They are used as lightweight fillers in composite materials such as syntactic foam and lightweight concrete. Microballoons give syntactic foam its lightweight, low thermal conductivity, and resistance to compressive stress that far exceeds that of other foams. These properties are exploited in the hulls of submersibles and deep-sea oil drilling equipment, where other types of foam would collapse. Hollow spheres of other materials create syntactic foams with different properties: ceramic balloons, for example, can produce a lightweight syntactic aluminium foam [2,6,10,11,12,13,14].

Hollow spheres also have various applications, including the storage and slow release of pharmaceuticals and radioactive tracers, as well as research on the controlled storage and release of hydrogen. Microspheres are also used in composites to fill polymer resins, imparting specific characteristics such as weight, wettability, and sealing properties to surfaces. Kai Zheng [15] stated that in recent years, porous bioactive glass micro/nanospheres (PBGSs) have emerged as attractive biomaterials in various biomedical applications, where these engineered particles provide suitable functions, ranging from tissue engineering to drug delivery.

Hong Wei et al. [16] used bioactive glass–ceramic microspheres with enhanced bioactivity for treating bone tissue. The electro-sprayed bioactive glass (ESBG) microspheres were 20 ± 5 μm in diameter and composed of four characteristic pores: <200 nm, 200–500 nm, 500–1000 nm and >1000 nm. The in vitro cytotoxicity was evaluated using the CCK-8 assay with MC3T3-E1 cells, demonstrating good biocompatibility. These results suggest that the ESBG microsphere is a promising material for bone tissue engineering. Also, Aleksandra et al. [17] used bioactive materials such as protein growth factors, peptides, amino acids, hormones, lipids, and flavonoids for bone regeneration.

Liu, X et al. [18] recently developed a three-dimensional (3D) scaffold technology for bone tissue engineering (BTE), which serves as an important therapeutic method for bone repairs. This can, however, cause long-term bone reconstruction because of the impediment associated with bone growth within the scaffolds and vascular infiltration.

Zhang Churu et al. [19] investigated the recent progress of using functionalized hollow hydroxyapatite microspheres (HHAMs) for biomedical applications. The HHAM is a unique type of mesoporous nanomaterial characterized by a specific internal cavity and hierarchical architecture, making it suitable for diagnosis, treatment, and theranostic technologies. The HHAM features promising biocompatibility, biodegradability, and a unique hollow core-shell structure, with good application prospects in biomedical fields such as drug delivery, controlled release, and diagnostic-guided clinical treatment. To unlock their full potential in the field of biomedicine and overcome the difficulties encountered in clinical treatment, versatile f-HHAMs have been developed. The utilization of HHAMs to transport specific therapeutic agents requires specific functional modifications to mitigate their side effects and achieve the desired therapeutic effect [19].

Several excellent reviews on hollow spheres, large hollow micro/nanospheres, glass beads, porous calcium phosphate glass microspheres, porous hydrogels and hydrogel microspheres, bioactive glass-based materials, and hollow mesoporous microspheres have been published [12,20,21,22,23,24,25,26,27,28,29]. However, there are few investigations on the use of hollow glass microspheres for biomedical applications. Therefore, this review aims to provide a comprehensive understanding of HGMs, encompassing their synthesis methods, characterization techniques, drug loading efficiency (DLE%) and diverse applications in regenerative medicine and biomedical fields. By examining the latest advancements and current challenges in the field, the review highlights the potential of hollow glass microspheres as versatile biomaterials that can contribute to the development of effective therapeutic strategies for tissue regeneration.

The method adopted for this review was the use of the Scopus search engine, Web of Science, and Google Scholar for selected articles using keywords such as “hollow glass microsphere”, “drug delivery”, “drug loading efficiency”, and “tissue engineering”.

### 1.1. Properties of Hollow Glass Microspheres

- Low density: HGMs are lightweight due to their hollow structure, resulting in high strength-to-weight ratios. This property makes them attractive for applications where weight reduction is critical, such as the aerospace and automotive industries.
- Low thermal conductivity: HGMs exhibit low thermal conductivity due to their hollow structure and small size, making them effective insulating materials in various thermal management applications.
- Insulation: The hollow interior of HGMs provides excellent thermal insulation properties, making them useful in applications requiring temperature control or insulation, such as building materials and protective coatings.
- High compressive strength: Despite their low density, HGMs possess high compressive strength (e.g., 10–120 MPa) based on their density, which makes them suitable for use as fillers or reinforcement agents in composites, structural materials and load-bearing for implants.
- Chemical inertness: Glassy shells of HGMs typically exhibit high chemical inertness, ensuring compatibility with a wide range of environments and applications. Also, their surface modification (e.g., silanization) can enhance the biocompatibility of HGM–protein interactions for application in regenerative medicine.
- Tailorable properties: The size, wall thickness, and composition of HGMs can be tailored during fabrication to meet specific requirements for different applications. These unique properties make HGMs highly versatile and applicable across various industries, including construction, transportation, electronics, and healthcare. In the medical field, their properties are being explored for applications such as drug delivery, tissue engineering, imaging, and diagnostics, offering promising avenues for innovation and advancement in medical technologies.

### 1.2. Importance of Exploring HGM Applications in Medical Sciences

**Figure 1:**
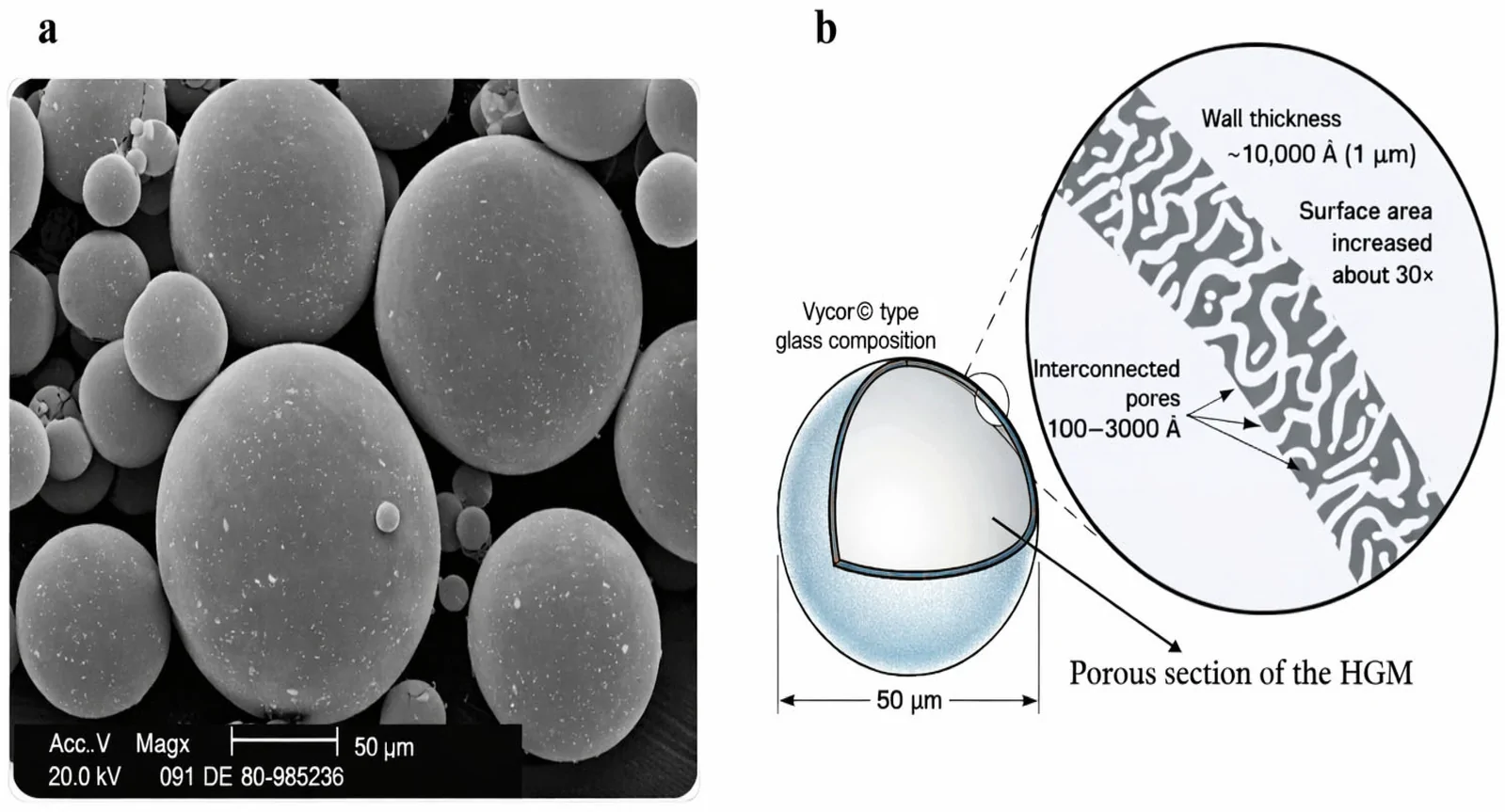
(**a**) Scanning electron microscopy of hollow glass microspheres (with a 50 μm scale bar); (**b**) schematic representations of the porous section of hollow glass microspheres for biomedical applications.

We are exploring the applications of hollow glass microspheres (HGMs) as a promising material in the field of biomedicine because they are bioinert materials, meaning they do not react with biological tissues or fluids. They remain stable and do not degrade, dissolve, or trigger immune responses when introduced into a biological environment [19,30]. To improve drug absorption and increase gastric retention, HGMs have been invented and spread in the clinical setup for certain patients using porous-wall HGMs. Figure 1a,b shows the scanning electron microscopy (SEM) images and the schematic representations of porous HGMs. The hollow or porous features of microspheres enable them to encapsulate fragile drugs, thereby protecting them from biological compounds that may interfere with drug availability. They often show promise for osteogenic applications. However, in vitro studies using MC3T3-E1 pre-osteoblasts highlight cytotoxicity risks that must be cautiously considered. Evidence suggests that certain dopants (e.g., Er^3+^, Tb^3+^), composites, or drug-loaded systems can impair cell viability or alter differentiation pathways, underscoring the need for balanced risk assessment alongside optimistic outcomes. Also, the dopant concentration must be tightly controlled to avoid adverse effects [31,32].

i. Drug Delivery Advancements: HGMs offer unique properties that can revolutionize drug delivery systems. Their hollow structure provides a reservoir for encapsulating drugs, allowing for controlled release kinetics and targeted delivery to specific tissues or cells. This capability can enhance therapeutic efficacy, reduce side effects, and improve patient compliance [24,33,34].
ii. Tissue Engineering and Regenerative Medicine: HGMs can be incorporated into scaffolds for tissue engineering applications. Their biocompatibility, tunable properties, and ability to enhance mechanical strength make them promising candidates for promoting tissue regeneration and wound healing. By facilitating cell adhesion, proliferation, and differentiation, HGM-based constructs have the potential to address challenges in tissue repair and organ transplantation [3,18,35].
iii. Imaging and Diagnostics Enhancement: In medical imaging, HGMs with size optimization of <50 µm can serve as contrast agents for various modalities, including ultrasound, MRI, and CT scans, because they reduce embolization risk during catheter delivery and can also be doped with paramagnetic or radiodense ions. Studies on embolic microspheres emphasize that elastic and small diameters (<50 µm) are essential for safe transcatheter delivery. HGMs must be engineered to deform slightly during catheter passage but return to spherical shape afterwards [34,36,37,38]. Their high echogenicity and ability to scatter or absorb radiation enable improved imaging resolution and diagnostic accuracy. Surface functionalization of HGMs can further enhance their targeting specificity and biocompatibility, paving the way for advanced diagnostic tools and imaging probes.
iv. Minimally Invasive Procedures: HGMs can enable the development of minimally invasive medical procedures by serving as carriers for therapeutic agents or imaging contrast agents. Their small size allows for delivery through fine-gauge needles or catheters, facilitating localized treatments and reducing the need for invasive surgeries.
v. Safety and Biocompatibility: Understanding the interactions between HGMs and biological systems is crucial for ensuring their safety and biocompatibility in medical applications. Comprehensive studies are necessary to evaluate cytotoxicity, immunogenicity, and long-term effects, thereby laying the groundwork for regulatory approval and clinical translation.
vi. Potential for Personalized Medicine: Tailoring the properties of HGMs, such as size, composition, and surface functionalization, can enable the development of personalized medical therapies. By customizing HGM-based platforms to meet the specific needs of individual patients, personalized medicine can be realized, leading to improved treatment outcomes and patient satisfaction.
vii. Interdisciplinary Collaboration: Exploring HGMs in medical sciences requires interdisciplinary collaboration between material scientists, chemists, biologists, engineers, and medical professionals. Such collaboration fosters innovation, accelerates research progress, and facilitates the translation of HGM-based technologies from the bench to the bedside. The drug loading efficiency (DLE %) of HGMs is generally lower compared to other materials, e.g., mesoporous silica nanoparticles (MSNs), which are specifically engineered with high surface area and tunable pore structures to maximize drug adsorption. HGMs are valued for their low density, bioinert nature, and ability to encapsulate drugs within their hollow core [33,39,40].

Table 1 presents a compilation of various materials and their corresponding biomedical applications, along with their drug loading efficiency (DLE %).

### 1.3. Chemical Structure of Hollow Glass Microspheres

Hollow glass microspheres are primarily composed of borosilicate glass, a material renowned for its low thermal expansion coefficient and chemical inertness. The chemical makeup typically includes silica (SiO_2_, 50–90%), alumina (Al_2_O_3_, 10–50%), and trace oxides like sodium (Na_2_O) and calcium (CaO). These components create a robust, lightweight structure with particle sizes ranging from 1 to 1000 micrometers and wall thicknesses of 1–2 micrometers. The borosilicate composition ensures high resistance to thermal shock and corrosion, making HGMs ideal for extreme environments. Also, their phase diagrams, which map out their equilibrium states (solid, liquid, gas, crystalline phases) as a function of temperature, pressure, and composition, contributed to HGMs being ideal for extreme environments [87,88,89]. The versatility and continuous development of HGMs highlight their importance and potential in medical treatments, particularly in bone tissue engineering and regeneration. Table 2 provides an overview of the chemical compositions of various HGMs, illustrating the diversity of these materials and their adaptability for various therapeutic purposes [90,91].

**Figure 2:**
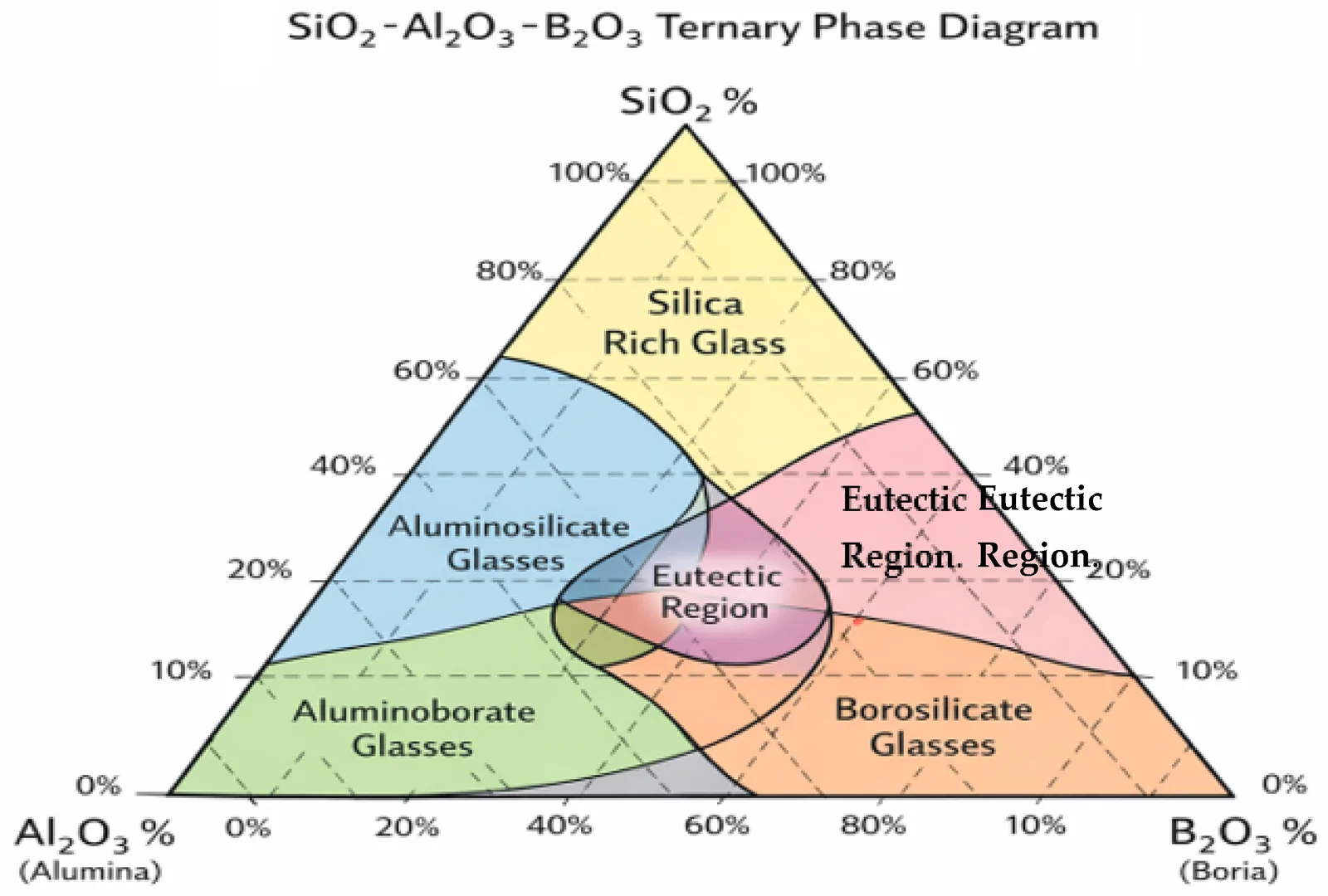
A schematic illustration of the SiO_2_-Al_2_O_3_-B_2_O_3_ ternary phase diagram.

Figure 2 shows the triangular composition space with silica (SiO_2_), alumina (Al_2_O_3_), and boria (B_2_O_3_) at the vertices, and the major glass-forming regions (silica-rich, aluminosilicate, borosilicate, aluminoborate, and boric-rich) separated by phase boundaries. At the center, the eutectic region is highlighted where the three components overlap [91,92].

**Table 2:** Constituents of hollow glass microsphere chemical elements by mass% [6,10,25,33,34,39,40,87,93,94,95].

Components	Symbol	Range	Biomedical Implications
Silica	${\mathrm{SiO}}_{2}$	50–90%	Promotes bone bonding; widely used in drug delivery and tissue engineering scaffolds.
Alumina	${\mathrm{Al}}_{2}O_{3}$	10–50%	Used in orthopedic implants, e.g., hip and knee prostheses.
Boron oxide	$B_{2}O_{3}$	0–12%	It promotes angiogenesis and wound healing; used in coatings for metallic implants.
Sodium oxide	${\mathrm{Na}}_{2}O$	0–0.1%	It increases solubility and ion release and contributes to pH changes that stimulate osteogenesis.
Potassium	$K_{2}O$	0–0.1%	Regulates dissolution rate and is generally safe in controlled release.
Lithium oxide	${\mathrm{Li}}_{2}O$	0–0.1%	It enhances glass bioactivity and stimulates bone regeneration.
Calcium oxide	CaO	0–10%	They are used in bone tissue engineering and remineralization.
Magnesium Oxide	MgO	0–10%	They enhance osteogenesis and enzymatic activity. They are beneficial in bone repairs and metabolic regulation.
Barium oxide	BaO	0–30%	They are used to adjust the refractive index and radiopacity in biomedical glasses.
Strontium oxide	SrO	0–30%	They are highly beneficial in bone regeneration and reduce osteoclast activity.

The hollow spherical geometry of HGMs is engineered to minimize material density while maximizing structural integrity. Each sphere contains a sealed cavity filled with inert gas (e.g., CO_2_ or nitrogen), which suppresses heat transfer via gas convection. The thin walls, often just 1% of the particle diameter, strike a balance between low density and mechanical strength. This design also enables efficient packing in composite materials, reducing voids and enhancing performance. However, the hollow glass microspheres obtained by such processes have a sufficient hollow degree to provide a lightening effect, a heat-insulating effect, or the like. Nevertheless, their average particle size is at least about 50 μm, and they contain particles having a maximum particle size exceeding 100 μm. Therefore, they cannot be used for applications requiring a smooth surface, those requiring a low dielectric constant, or those needing a composite material with a restricted thickness [87].

### 1.4. Types of Hollow Glass Microspheres

Hollow glass microspheres (HGMs) are primarily categorized by their chemical composition and physical properties, including density, particle size, and compressive strength, which determine their specific applications. HGMs achieved high purity performance metrics with a low dielectric constant of ε_r < 4 because microspheres contain air-filled cores, and since air has ε_r ≈ 1, the effective dielectric constant of the composite drops significantly. This improves their impact on bioelectronics, such as reducing capacitive losses, stable high-frequency signals, withstanding sterilization without crystallization, safe for implantable and wearable devices, and preventing dielectric degradation in biological fluid [96]. Table 3 is composed of different series of HGMs, e.g., H series—H20, K series—K15, T series—T60, and M series—M40 [94,97,98,99,100].

### 1.5. Structural and Compositional Characterization of Hollow Glass Microspheres

The structural and compositional characterization of hollow glass microspheres (HGMs) involves techniques to determine their morphology, chemical composition, and key physical properties, such as density and crush strength [105,106,107].

#### 1.5.1. Structural Characterization

Structural characterization focuses on the physical form, dimensions, and integrity of the microspheres:

- Morphology: Field Emission Scanning Electron Microscopy (FE-SEM) or Environmental Scanning Electron Microscopy (ESEM) is used to observe the spherical nature, surface smoothness, and the presence of pores or defects.
- Particle Size and Distribution: The diameter (typically 10 to 300 µm) and size distribution are determined using techniques like laser particle size analyzers or image analysis systems [5,108].
- Wall Thickness: The wall thickness (ranging from sub-micron to a few micrometers) is often observed from the cross-section of a fractured microsphere using SEM images [5,13]. HGMs are typically fabricated via flame-spraying or flame-fusion techniques. The flame temperature directly influences the viscosity of the molten glass. This means that the lower the viscosity, the thinner the walls and the larger the diameter of the HGMs, which increases fragility and enhances drug release kinetics due to faster diffusion. HGMs can encapsulate drugs either by diffusion into the hollow cavity or by surface adsorption followed by sealing. Also, adjusting fabrication parameters can align HGMs with specific biomedical needs, e.g., chemotherapy (i.e., rapid release) against chronic disease management (i.e., sustained release) [109,110].
- Internal Structure/Porosity: Examining cracked microspheres via SEM confirms the hollow structure and assesses internal porosity. However, SEM is limited in that it cannot quantify internal gas composition without the use of Focused Ion Beam-Scanning Electron Microscopy (FIB-SEM). The FIB-SEM is a powerful dual-beam technique used in materials science, nanotechnology, and biomedical engineering for both imaging and sample preparation. It can confirm microstructural uniformity in amorphous coatings or composites and helps correlate thermal stability with microstructural changes [8,9].
- Density: A true density tester or the drainage method is used to measure the bulk and true particle density, which are critical for their lightweight applications.
- Mechanical Properties: Crush strength and compressive properties are analyzed using a universal testing machine (e.g., Instron) to determine the pressure at which the microspheres fail. This property is directly related to wall thickness and overall structural integrity.
- Thermal Properties: Techniques like Differential Scanning Calorimetry (DSC) and thermogravimetric analysis (TGA) are used to determine the glass transition temperature, thermal stability, and thermal conductivity. A thermal constant analyzer can measure thermal conductivity.
- Raman Spectroscopy: Raman spectroscopy is well-known as a nondestructive technique used for vibrational, rotational, and other low-frequency modes in molecules and provides the structural fingerprints of materials for identification. It probes vibrational modes of Si-O bonds and structurally confirms the purity and amorphous silica nature of HGMs, correlating with low dielectric constants (ε_r < 4). Also, in phase identification of HGMs, it distinguishes between amorphous silica (broad bands) and crystalline phases (sharp peaks), which helps in HGM biomedical process optimization [111].

#### 1.5.2. Compositional Characterization

Compositional characterization identifies the chemical makeup of the glass material:

- Chemical Composition: Inductively Coupled Plasma Atomic Emission Spectrometry (ICP-AES) is often used to determine the elemental composition of the glass frit raw material and the final product. HGMs are typically composed of soda-lime or borosilicate glass, with the main components being silicon dioxide (SiO_2_) and calcium oxide (CaO).
- Crystalline Structure: X-ray Diffraction (XRD) is used to determine if the glass is amorphous (which it typically is) or whether any crystalline phases have formed during synthesis or post-processing (e.g., in glass-ceramics microspheres).
- Chemical Bonding/Functional Groups: Fourier Transform Infrared Spectroscopy (FTIR) is employed to analyze chemical bonds and functional groups, which is useful for characterizing surface modifications or the presence of specific additives/blowing agents.
- Chemical Stability/Solvent Resistance: The resistance to various solvents and chemicals is assessed to ensure stability in different application environments.

## 2. Fabrication Method of Hollow Glass Microspheres

### 2.1. Overview of Common Fabrication Techniques

Common fabrication techniques for hollow glass microspheres (HGMs) (e.g., glass powder method, sol-gel, spray drying, template-assisted methods) involve processes that allow precise control over their size, morphology, and properties. Here is an overview of some commonly used fabrication methods:

#### 2.1.1. Glass Powder Method

The glass powder method involves melting borosilicate glass, atomizing it into droplets, and rapidly cooling these droplets to form hollow spheres. This process requires precise temperature control to ensure uniform wall thickness and prevent defects from occurring.

#### 2.1.2. Sol-Gel Method

**Figure 3:**
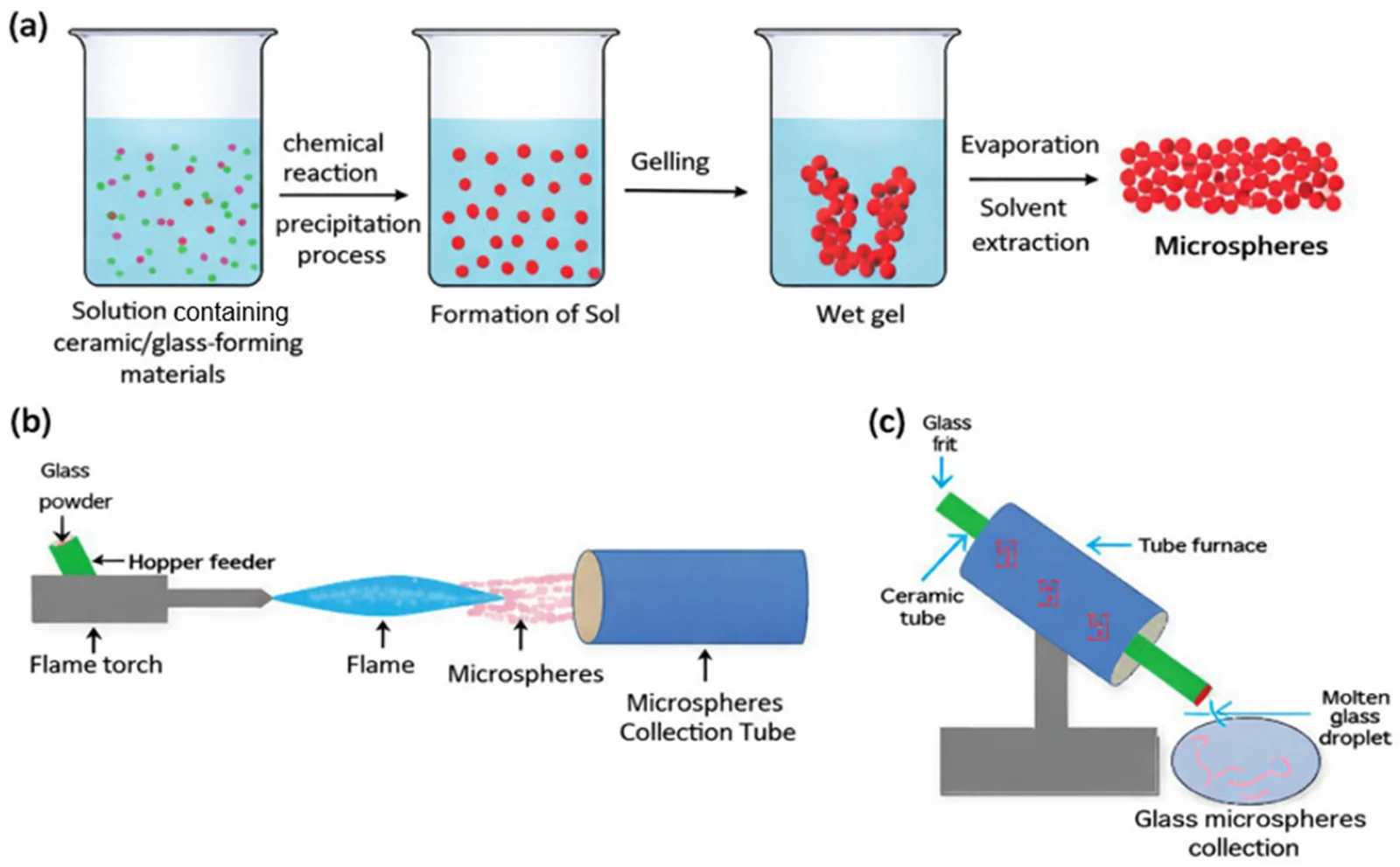
Glass microsphere production by (**a**) sol-gel, (**b**) flame spheroidization and (**c**) tube furnace methods (Adapted with permission. 2019 Journal of Science and Engineering [2]).

This process involves converting precursor solutions (typically xerogel) into a gel phase, followed by drying and heat treatment to form glassy particles. Typically, a template material (e.g., polymer microspheres or surfactant micelles) is used to create the hollow structure. The gel is typically dried under controlled conditions to prevent the collapse of its hollow structure. HGMs are manufactured from a sol-gel-derived sodium borosilicate glass with a composition suitable for photo-enhanced hydrogen diffusion in hydrogen storage applications. The heat-treated xerogel is suspended and doped with iron sulphate or iron chloride to provide the transition metal and bubbling agent, and then spray-dried. The resulting granules are subsequently flame-sprayed in an oxy–propane flame to obtain glass microspheres. *N*–hexadecyl tri-methyl ammonium chloride is incorporated into selected suspensions before spray-drying to achieve hollow spray-dried particles, which promise improved HGMs. The agglomeration of the spray-dried granules results in a lowered flowability of the granules, reducing the effectiveness and quality of HGM manufacture. Figure 3 illustrates the manufacturing process of microspheres using the gel method [2].

**Figure 4:**
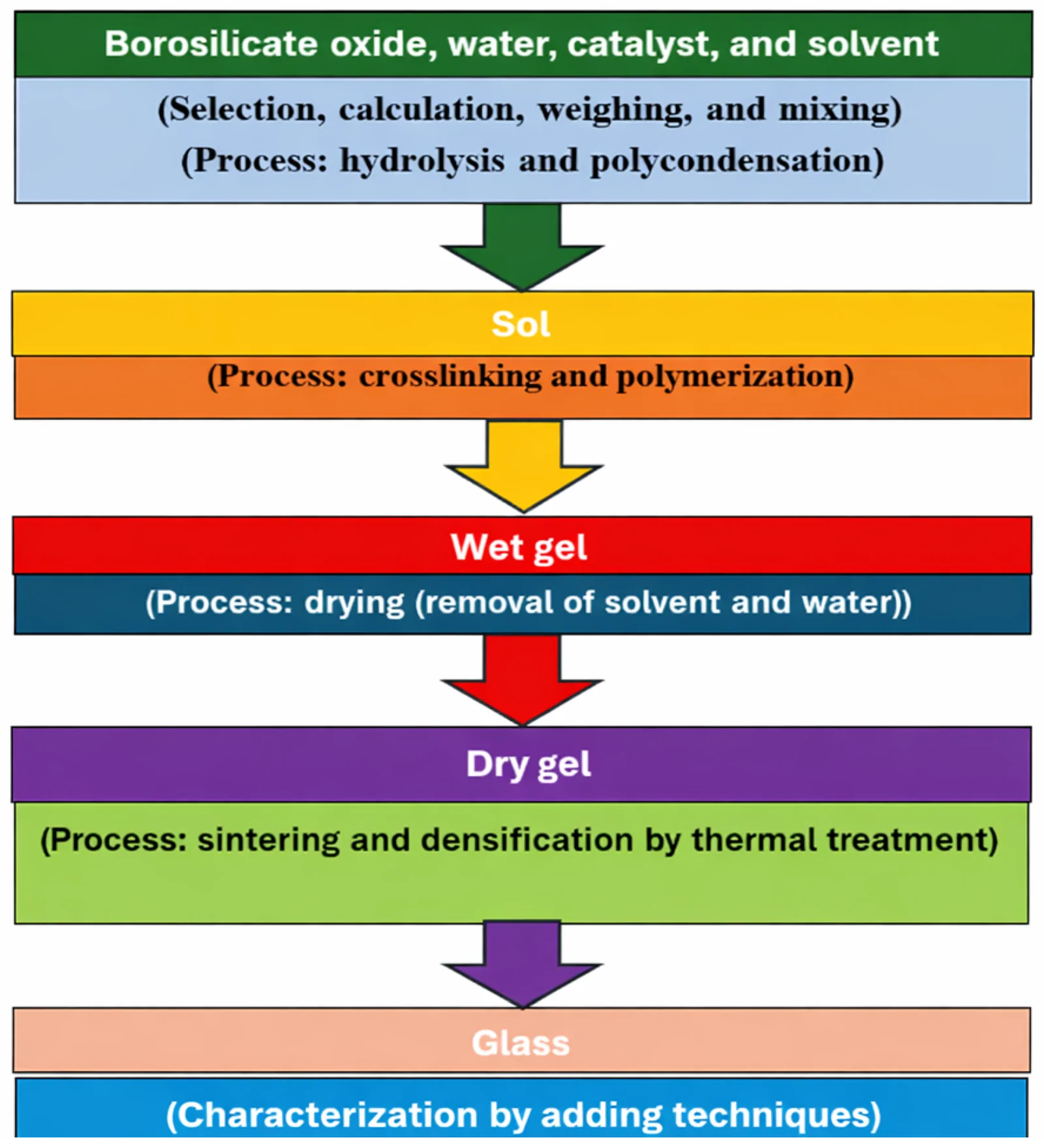
Schematic representation of sol-gel techniques.

This method allows for precise control over the size and composition of the microspheres. Figure 4 presents a diagrammatic representation of the steps involved in preparing the glass using the sol-gel method. The process begins with the selection, calculation, weighing, and mixing of alkoxides, water, catalysts, and solvents. Hydrolysis and polycondensation reactions result in the formation of a sol, which undergoes crosslinking and polymerization to develop a gel-like network [90]. The resulting wet gel is then subjected to a process in which solvents and water are removed to form a dry gel.

This dry gel is subsequently sintered and densified through thermal treatment to obtain the final glass structure. The synthesized glass is then characterized using various analytical techniques to assess its structural and functional properties.

#### 2.1.3. Spray Drying

**Figure 5:**
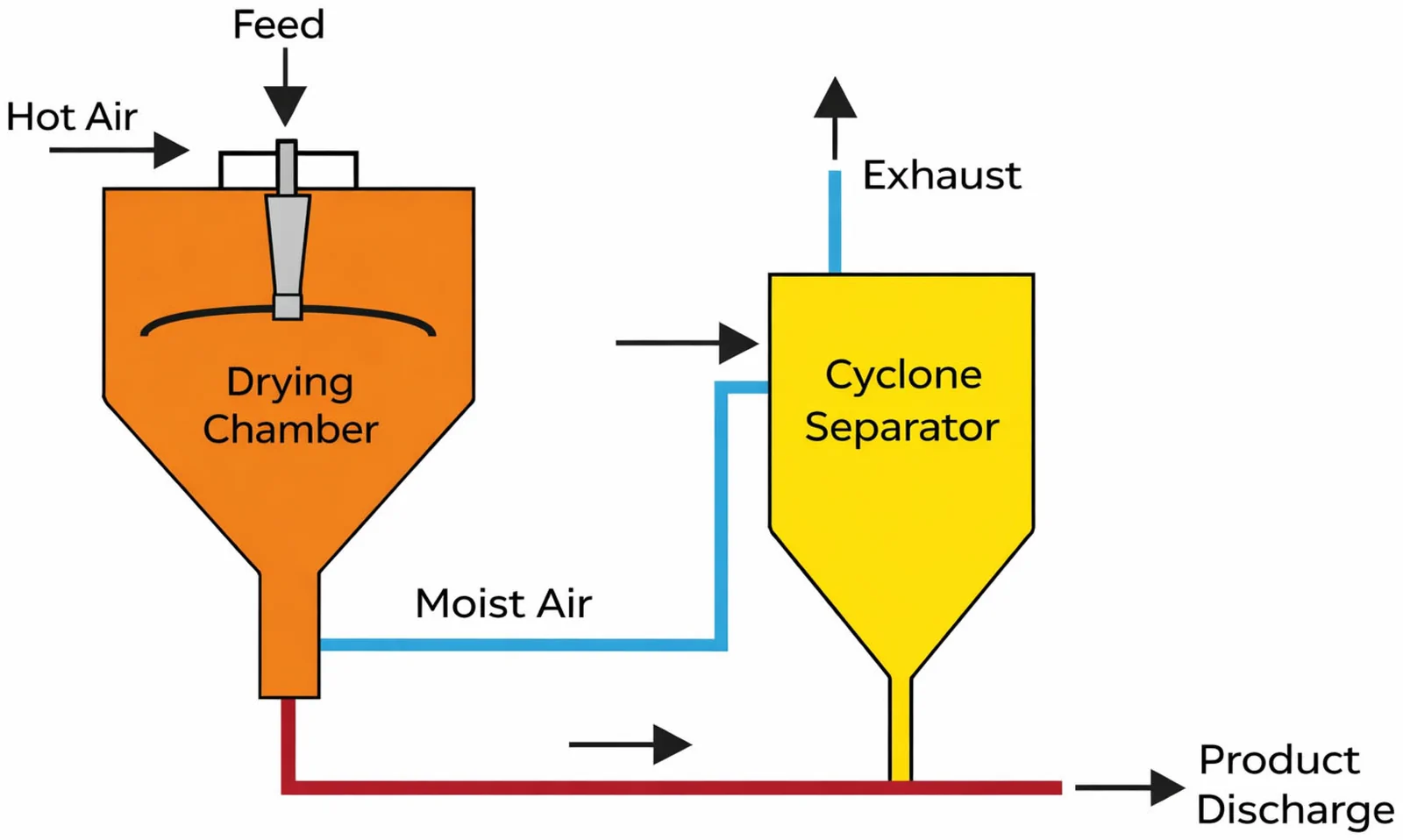
Spray-drying technique (Adapted with permission. 2023 International Journal of Research and Analytical Reviews [4]).

This was utilized to create a polymeric blended microsphere containing the medication ketoprofen. It involves mixing the core material with the liquid coating material and then spraying the combination into the environment for coating solidification, followed by rapid solvent evaporation. Organic solutions of poly (epsilon-caprolactone) (PCL) and cellulose acetate butyrate (CAB) in various weight ratios, as well as ketoprofen, were produced and sprayed under various experimental circumstances to produce drug-loaded microspheres. This is a quick process, although the crystallinity may be lost due to the rapid drying, as illustrated in Figure 5.

This method involves atomizing a solution or suspension of glass precursors into fine droplets, which are then dried using hot air or inert gas. As the solvent evaporates, solid microspheres are formed, often with a hollow interior. The morphology and size distribution of the microspheres can be controlled by adjusting parameters such as feed composition, drying temperature, and airflow rate. Spray drying is a scalable and relatively straightforward technique suitable for large-scale production [4,23].

#### 2.1.4. Template-Assisted Methods

This process utilizes sacrificial templates to create the hollow structure of the microspheres. Templates can be solid particles, emulsion droplets, or self-assembled structures (e.g., colloidal crystals). The glass precursor is deposited onto the templates, which are subsequently removed (e.g., by dissolution or combustion) to leave behind hollow microspheres. This method enables precise control over the size, shape, and porosity of the microspheres by selecting suitable templates and adjusting the processing conditions [23].

#### 2.1.5. Emulsion Templating

**Figure 6:**
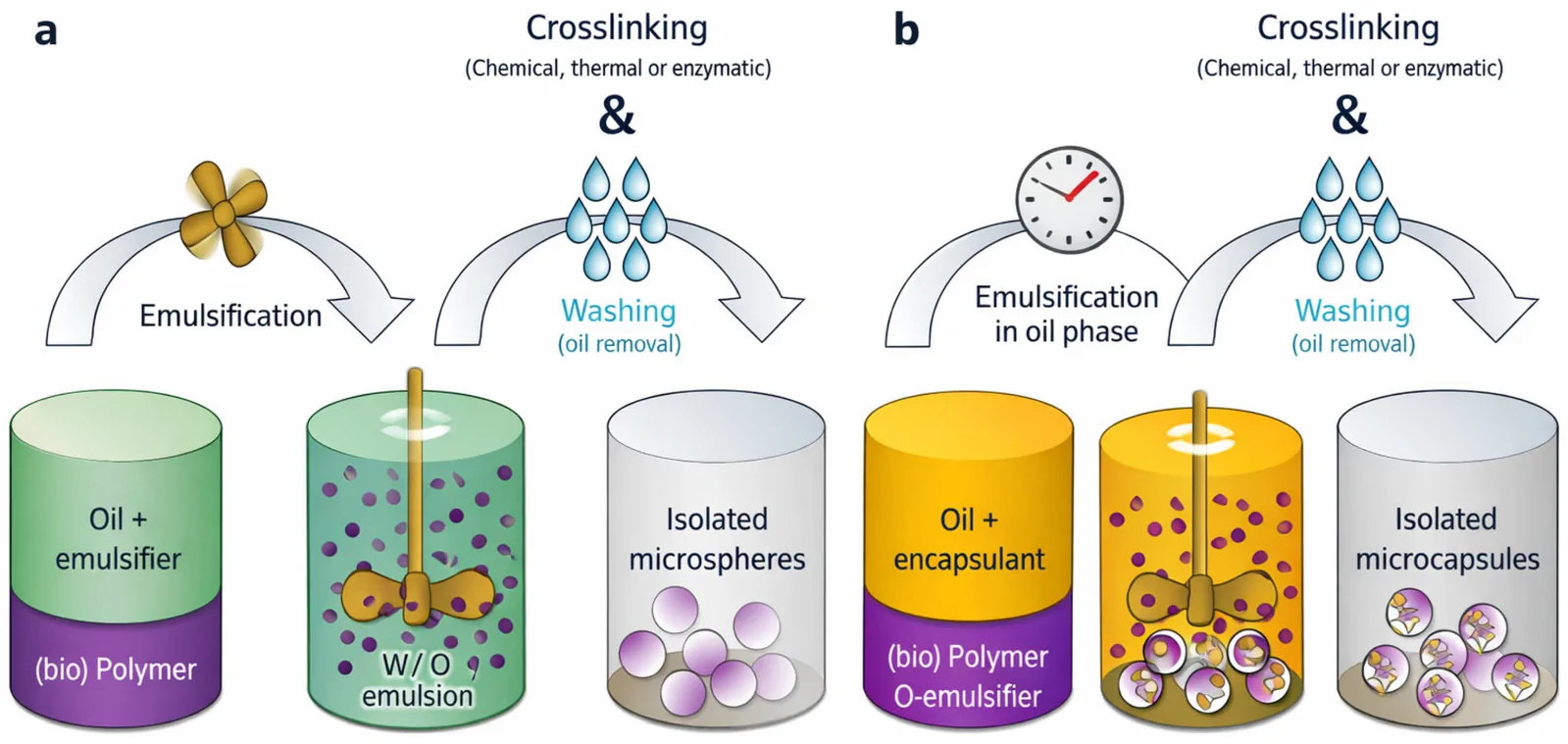
(**a**) Single-emulsion technique and (**b**) double-emulsion technique (Adapted with permission. 2023 International Journal of Research and Analytical Reviews [4]).

This process involves emulsifying a mixture of glass precursor and surfactants in a continuous phase. The emulsion droplets serve as templates for the hollow structure, and the glass precursor is polymerized or solidified within the droplets. This process can be either a single-emulsion or a double-emulsion technique. Single emulsions are either o/w or w/o emulsions. Chemical crosslinkers are utilized in the single-emulsion approach for crosslinking. Crosslinkers such as diacid chloride, glutaraldehyde, formaldehyde, and others are utilized. In the double-emulsion technique, both natural and synthetic polymers are used for the synthesis of microspheres. This involves the formation of multiple emulsions, specifically the double-emulsion type w/o/w. This technique is suitable for proteins, peptides, vaccines, and water-soluble drugs [4]. Figure 6a,b illustrates the process of single- and double-emulsion techniques. After solidification, the surfactants are typically removed by washing or calcination to yield hollow glass microspheres. This method allows for the synthesis of monodisperse microspheres with tunable sizes and shell thicknesses [4,23].

#### 2.1.6. Sintering of Hollow Glass Microspheres

In this process, the synthesis of hollow glass microspheres is followed by sintering at high temperatures to convert them into glassy microspheres. Glass microspheres can be synthesized using methods such as emulsion polymerization or microfluidic techniques. Sintering of glass particles can soften and fuse at elevated temperatures without decomposition because their thermal stability is very high. They are stable up to 600–1000 °C and can retain their shape if sintered carefully [82,106,112]. This method offers flexibility in controlling the composition and properties of the microspheres by selecting appropriate polymer precursors.

These fabrication techniques offer diverse approaches for producing HGMs with tailored properties, suitable for various applications in industries such as aerospace, automotive, construction, and, increasingly, the medical sciences. Each method has its advantages and limitations, and the choice of technique depends on factors such as desired particle size, morphology, composition, and scalability requirements [112].

### 2.2. Factors Influencing the Size, Shape, and Properties of HGMs

The size, shape, and properties of hollow glass microspheres (HGMs) can be influenced by various factors, including the fabrication method, precursor composition, processing conditions, and post-treatment steps [95,98,101,106]. Here is an overview of the key factors:

#### 2.2.1. Fabrication Method

Different fabrication techniques, such as sol-gel, spray drying, template-assisted methods, and emulsion templating, result in HGMs with distinct sizes, shapes, and properties. Each method offers unique control over parameters such as particle size distribution, shell thickness, and hollow structure.

#### 2.2.2. Precursor Composition

The composition of the glass precursor or starting materials used in HGM synthesis significantly impacts the final properties of the microspheres. Variations in precursor composition, including the type of glass-forming components (e.g., silica, borosilicate), dopants, and additives, can influence factors such as mechanical strength, thermal stability, and chemical reactivity.

#### 2.2.3. Processing Conditions

Parameters such as temperature, pressure, pH, reaction time, and stirring rate during the fabrication process play crucial roles in determining the size, morphology, and properties of HGMs. Control over these processing conditions enables the optimization of particle size, shell thickness, and structural integrity.

#### 2.2.4. Template Properties

In template-assisted methods, the properties of the sacrificial templates (e.g., size, shape, porosity) directly influence the size, shape, and porosity of the resulting HGMs. The selection of appropriate templates and control over template properties enable precise control over the morphology and structure of the microspheres.

#### 2.2.5. Post-Treatment Steps

Post-treatment steps such as annealing, calcination, etching, and surface modification can further alter the size, morphology, and properties of HGMs. Annealing or calcination processes can enhance the mechanical strength and thermal stability of the microspheres, while etching can control surface roughness and porosity.

#### 2.2.6. Additives and Surface Functionalization

Incorporation of additives or surface functionalization agents during synthesis or post-treatment can impart specific properties to HGMs. Additives such as surfactants, stabilizers, or dopants can modify properties such as dispersibility, surface charge, or optical properties.

#### 2.2.7. Scale-Up Considerations or Scalability and Clinical Translation Challenges

When considering synthesis methods, such as flame spraying and sol-gel, that have been extensively studied for their influence on hollow glass microsphere (HGM) properties, including size distribution, porosity, and surface chemistry, their clinical translation requires careful consideration of scalability and regulatory compliance. The scalability of fabrication methods is essential for the large-scale production of HGMs with consistent size, shape, and properties. Factors such as cost-effectiveness, throughput, and reproducibility need to be considered when scaling up production processes.

Understanding and optimizing these factors are crucial for tailoring HGMs to meet specific requirements for various applications, including lightweight fillers, insulating materials, drug delivery carriers, and tissue engineering scaffolds. Researchers continually strive to refine fabrication techniques and control the parameters of HGMs to achieve the desired properties for various applications. Some of the critical challenges emerging from a pharmaceutical engineering standpoint include (i) ensuring proper yield optimization because the translational success depends on process intensification strategies that maximize yield without compromising structural integrity and (ii) good manufacturing practice (GMP) because frameworks demand strict control of raw materials, solvents, and processing conditions. Sol-gel routes frequently involve organic solvents and catalysts that must be fully removed to guarantee biocompatibility, while flame spraying requires reproducibility and contamination control at elevated temperatures. Attaining GMP standards necessitates validated protocols for sterility, traceability, and reproducibility (ISO 10993-1:2025 [113]).

## 3. Drug Delivery Applications

### 3.1. Utilization of HGMs as Drug Carriers for Controlled Release and Targeted Delivery

The utilization of hollow glass microspheres (HGMs) as drug carriers for controlled release and targeted delivery offers several advantages in the field of pharmaceuticals. Here is an overview of how HGMs are employed in drug delivery systems (see Table 1) [34,39,72].

#### 3.1.1. Encapsulation of Drugs

HGMs provide a hollow interior that can serve as a reservoir for loading various types of drugs, including small molecules, proteins, peptides, and nucleic acids. Drugs can be encapsulated within the hollow cores of HGMs via adsorption, absorption, or chemical conjugation, depending on the drug’s physicochemical properties and the surface properties of the microspheres to improve their biocompatibility, the release kinetics, and in vivo efficacy [114,115].

#### 3.1.2. Controlled Release Kinetics

The hollow structure of HGMs enables the controlled release of encapsulated drugs over time. Factors such as the thickness of the glass shell, the size of the pores, and the physicochemical properties of the drug influence the release kinetics [114,115]. By adjusting these parameters, researchers can modify drug release profiles to achieve desired therapeutic outcomes, including sustained, pulsatile, or triggered release in response to specific stimuli (e.g., pH, temperature, or enzymatic activity) [31].

#### 3.1.3. Protection of Drugs

HGMs protect encapsulated drugs from enzymatic degradation, pH fluctuations, and other environmental factors. The glassy shell of HGMs acts as a barrier, shielding the encapsulated drugs from external influences and enhancing their stability during storage and transport.

#### 3.1.4. Enhanced Bioavailability

HGMs can improve the bioavailability of poorly soluble drugs by increasing their solubility and dissolution rates. The high surface area-to-volume ratio of HGMs facilitates rapid dispersion and dissolution of encapsulated drugs, thereby enhancing absorption and therapeutic efficacy.

#### 3.1.5. Targeted Delivery

Surface modification of HGMs with targeting ligands (e.g., antibodies, peptides, aptamers) enables specific recognition and binding to target cells or tissues. Targeted HGM-based drug delivery systems can selectively deliver drugs to diseased sites while minimizing off-target effects and reducing systemic toxicity. Active targeting strategies can enhance drug accumulation at the desired site of action, improving therapeutic outcomes and reducing the required dosage [31].

#### 3.1.6. Multifunctional Platforms

HGMs can be integrated into multifunctional drug delivery platforms that combine therapeutic agents with imaging agents, diagnostic probes, or stimuli-responsive components. Such multifunctional systems enable simultaneous drug delivery and monitoring of therapeutic responses, allowing for real-time feedback and personalized treatment strategies. Overall, HGM-based drug delivery systems offer versatile platforms for achieving controlled release, targeted delivery, and enhanced therapeutic efficacy of a wide range of drugs. Continued research into optimizing HGM properties, surface functionalization strategies, and formulation techniques holds promise for developing innovative drug delivery solutions with improved patient outcomes.

### 3.2. Surface Modification Strategies to Enhance Biocompatibility and Loading Efficiency

Surface modification of hollow glass microspheres (HGMs) is essential for enhancing their biocompatibility and loading efficiency in drug delivery applications. Here are some surface modification strategies commonly employed for this purpose:

#### 3.2.1. Surface Functionalization with Biocompatible Polymers

Coating HGMs with biocompatible polymers, such as polyethene glycol (PEG), polyvinyl alcohol (PVA), or chitosan, enhances their biocompatibility and reduces nonspecific interactions with biological components. These polymers create a hydrophilic surface layer that minimizes protein adsorption, reduces immune recognition, and enhances colloidal stability in biological fluids.

#### 3.2.2. Introduction of Targeting Ligands

Conjugation of targeting ligands (e.g., antibodies, peptides, aptamers) onto the surface of HGMs enables specific recognition and binding to target cells or tissues. Targeting ligands enable active targeting of drug-loaded HGMs to diseased sites, thereby enhancing drug accumulation and therapeutic efficacy while minimizing off-target effects.

#### 3.2.3. Surface Charge Modification

Altering the surface charge of HGMs by functionalizing them with charged molecules or polymers (e.g., cationic or anionic surfactants) can influence their interactions with biological membranes and cells. Surface charge modification can enhance cellular uptake, promote endosomal escape, and improve intracellular delivery of drugs encapsulated within HGMs.

#### 3.2.4. Mesoporous Coatings

The deposition of mesoporous coatings on the surface of HGMs creates additional pore structures that can enhance loading efficiency and enable controlled drug release. Mesoporous coatings offer a high surface area for drug adsorption, facilitate drug diffusion into the porous structure, and enable sustained release kinetics.

#### 3.2.5. Crosslinking and Surface Engineering

Crosslinking agents, such as glutaraldehyde or silanes, can be used to functionalize the surface of HGMs, thereby improving their stability, mechanical strength, and resistance to degradation. Surface engineering techniques, including plasma treatment, chemical grafting, or layer-by-layer assembly, enable precise control over surface properties and functionalities.

#### 3.2.6. Incorporation of Stimuli-Responsive Materials

The introduction of stimuli-responsive materials onto the surface of HGMs enables triggered drug release in response to specific environmental stimuli (e.g., pH, temperature, light, enzymes). Stimuli-responsive coatings or nanoparticles can be designed to undergo conformational changes or disassembly in response to external cues, facilitating on-demand drug release at the target site.

#### 3.2.7. Bioconjugation Chemistry

Bioconjugation techniques, such as click chemistry, thiol-ene reactions, and amine-carboxyl coupling, enable site-specific attachment of biomolecules, drugs, or targeting ligands to the surface of HGMs. Bioconjugation chemistry offers precise control over the orientation, density, and functionality of surface-modified moieties, thereby optimizing drug loading efficiency and biological activity. By employing these surface modification strategies, researchers can tailor the properties of HGMs to meet specific requirements for drug delivery applications, including improved biocompatibility, enhanced targeting capability, and efficient loading and release of therapeutic agents.

Several case studies demonstrate the successful utilization of hollow glass microspheres (HGMs) in drug delivery systems, showcasing their potential in enhancing therapeutic efficacy, controlled release, and targeted delivery. Here are a few examples:

##### Paclitaxel-Loaded HGMs for Cancer Therapy

- Hollow glass microspheres were loaded with paclitaxel, a widely used chemotherapeutic agent, through a solvent evaporation method.
- The drug-loaded HGMs exhibited sustained release of paclitaxel over several days, with enhanced cytotoxicity against breast cancer cells compared to free paclitaxel.
- In vivo studies in tumor-bearing mice demonstrated improved antitumor efficacy and reduced systemic toxicity of paclitaxel when delivered using HGMs, highlighting their potential as effective drug carriers for cancer therapy.

##### Bone-Targeted Drug Delivery Using Bisphosphonate-Functionalized HGMs

- In the studies published in the journal ACS Biomaterials [17,42], a bone-targeted drug delivery system using bisphosphonate-functionalized HGMs was developed.
- Hollow glass microspheres were surface-modified with alendronate, a bisphosphonate drug known for its affinity to bone tissue.
- The alendronate-functionalized HGMs exhibited enhanced binding affinity to hydroxyapatite, a significant component of bone, enabling targeted delivery of therapeutic agents to bone tissue.
- Drug-loaded HGMs functionalized with alendronate demonstrated improved retention and sustained release of drugs in bone tissue, making them promising candidates for the treatment of bone-related diseases such as osteoporosis and bone metastases.

##### Dual-Drug Delivery System for Combination Therapy

- Hollow glass microspheres were loaded with two chemotherapeutic drugs, doxorubicin and camptothecin, through a co-precipitation method.
- The dual-drug-loaded HGMs exhibited synergistic cytotoxic effects against cancer cells in vitro, with enhanced antitumor efficacy compared to single-drug formulations.
- In vivo studies in tumor-bearing mice demonstrated improved tumor regression and prolonged survival rates when treated with the dual-drug-loaded HGMs, highlighting the potential of HGMs for combination therapy in cancer treatment [72].

## 4. Conclusions and Future Outlook

Hollow glass microspheres (HGMs) are used in biomedical applications for controlled drug delivery, acting as carriers for drugs, especially for targeted cancer therapies (like chemotherapy) or oral medications needing longer gastric retention, improving efficacy and reducing side effects. The potential of microspheres in drug delivery has been investigated since the 1980s, but their use in biomedical applications has been in the limelight for over two decades.

This study demonstrates the versatility and effectiveness of HGM characterization, fabrication processes, and properties as drug delivery carriers across various therapeutic applications, including cancer therapy, bone targeting, and combination chemotherapy. Continued research efforts to optimize HGM-based drug delivery systems hold promise for addressing current challenges in drug delivery and improving patient outcomes across diverse disease conditions.

Future outlook on this research can focus on how to reduce large water intake for oral administration of drugs to ensure floating and proper function. Also, experimental work on in vivo studies on HGM-doped scaffolds for bone regeneration could be considered to align with the current trends in theranostics (i.e., combining diagnostic imaging and targeted therapy into a single, cohesive intervention). However, drug stability must be maintained because some drugs are unstable in gastric fluids.

## Figures and Tables

**Table 1:** Different glass microspheres and other materials, with their biomedical applications.

Materials	Biomedical Applications	DLE (%)	Ref.
Porous calcium phosphate glass microspheres (PCPGMs)	Orthobiologics: This involves the use of cell-based therapies and biomaterials to enable the body to repair and regenerate musculoskeletal tissues.	30–50	[12,41]
Nanofibrous microspheres (NFMSs)	Bone regeneration: Using extracellular matrix architecture.	50–70	[42,43]
Bioactive glass-based materials	Bone regeneration, orthopedic and dental applications: The use of innovative biomaterials to allow the coordination and well-controlled repair of bone fractures and bone loss, designed to reduce the need for autologous or allogeneic bone grafts.	20–45	[17,28,35,44,45,46,47,48,49,50,51,52,53]
Hybrid composites of poly(methylmethacrylate)/poly(ɛ-caprolactone) (PMMA/PCL)	Tissue engineering: The formation of tissue and regeneration using artificial scaffolds designed to direct tissue growth.	15–30	[30,54,55,56,57]
Immunomodulatory biomaterials	Therapy for chronic inflammatory disease	20–50	[58,59]
Structured scaffolds; piezoelectric materials, nanomaterial-assisted theranosis	Bone repair: They utilized multiple materials for bone repair and regeneration through physiological, biochemical, electrical, and mechanical processes.	30–45	[60,61,62,63,64,65]
Reinforced silk/magnesium filament	Peripheral nerve repair: They required artificial nerve grafts, including nerve guidance conduit (NGCs) with biocompatibility, suitable mechanical properties and topography to guide axon growth.	20–35	[66,67]
Silk	Wound healing and tissue engineering	20–40	[68,69]
Nanocarbons	Cancer diagnostics and therapeutic tools	40–80	[70,71]
Mesoporous silica nanoparticles (MSNs)	Drug delivery	40–60	[72,73]
Layered Double Hydroxides (LDHs)	Cancer diagnosis and therapy, tissue engineering and biosensors,	30–60	[74,75,76]
Porous polyvinyl alcohol (PVA) scaffold	Bone tissue engineering	20–35	[77,78,79]
Hollow glass microspheres (HGMs)	Drug delivery, tissue engineering and implants, diagnostic devices, and nanocarriers in biotechnology	40–80	[33,34,39,40,80,81,82,83,84,85,86]

**Table 3:** Hollow glass microsphere classifications [1,10,93,94,97,98,99,100,101,102,103,104].

Classification by Composition	Classification by Physical Properties and Performance	Classification by Application
Soda-Lime_Borosilicate Glass	Density: Ranges from very low (e.g., 0.10 g/cm^3^) to moderate (e.g., 0.60 g/cm^3^)	Lightweight fillers: Used in plastics, rubber, and synthetic materials
Aluminosilicate Glass	Compressive strength (e.g., 10 MPa for low densities, and up to 30 MPa for high densities): Used for applications under high pressure	Thermal and acoustic insulation
High-Purity Silica Glass	Particle size: Its diameters generally range from 2 to 200 micrometers	Deep-sea buoyancy materials: High-strength types for critical components
Special Formulation Glass	Surface modification: HGM with special surface treatment	Oil and gas industry: Used in drilling fluids and cementing

## Data Availability

No new data were created or analyzed in this study. Data sharing is not applicable to this article.

## Abbreviations

The following abbreviations are used in this manuscript:

HGM	Hollow glass microspheres
PQ	Philadelphia quartz
PBGSs	Porous bioactive glass micro/nanospheres
ESBG	Electro-sprayed bioactive glass
HHAM	Hollow hydroxyapatite microspheres
SEM	Scanning electron microscopy
PMMA/PCL	Poly(methyl-methacrylate)/poly(ɛ-caprolactone)
FE-SEM	Field emission scanning electron microscopy
ESEM	Environmental scanning electron microscopy
DSC	Differential scanning calorimetry
TGA	Thermogravimetric analysis
FTIR	Fourier transform infrared spectroscopy
XRD	X-ray diffraction
